# Analysis of phenolic compounds and immunomodulatory activity of areca nut extract from Aceh, Indonesia, against *Staphylococcus aureus* infection in Sprague-Dawley rats

**DOI:** 10.14202/vetworld.2020.134-140

**Published:** 2020-01-18

**Authors:** Liza Meutia Sari, Rachmi Fanani Hakim, Zaki Mubarak, Andriyanto Andriyanto

**Affiliations:** 1 Department of Oral Medicine, Faculty of Dentistry, University of Syiah Kuala, Banda Aceh, Indonesia; 2 Department of Oral Biology, Faculty of Dentistry, University of Syiah Kuala, Banda Aceh, Indonesia; 3 Veterinary Teaching Hospital, Faculty of Veterinary Medicine, Bogor Agricultural University, Bogor, Indonesia

**Keywords:** areca nut extract, catechin, immunomodulatory activity, phytochemical content

## Abstract

**Aim:**

The aim of the study was to investigate the immunomodulatory activity of areca nut extract. The phytochemical content and phenolic composition of the extract were also determined.

**Materials and Methods:**

An extract of areca nut was prepared using 96% ethanol and subsequently screened for phytochemical content using a high-performance liquid chromatography (HPLC) method. The immunomodulatory activity of the extract was tested in 35 Sprague-Dawley rats, divided into four groups: One control group and three experimental groups in which the rats received 500, 1000, or 1500 mg/kg of oral areca nut extract biweekly (BW). The extract was orally administered 14 days before the intraperitoneal challenge with *Staphylococcus aureus* (1×10^8^ CFU/mL). On the 14^th^ day of the experiment, rats in all the four groups were sacrificed. Measurement of the levels of red blood cells, hematocrit (Hct), hemoglobin (Hb), white blood cells (WBCs), lymphocytes, monocytes, neutrophils, basophils, eosinophil, and macrophages were recorded. The activities of serum glutamate oxalate transaminase, serum glutamate pyruvate transaminase, urea, and creatinine were also determined.

**Results:**

Areca nut was found to contain an alkaloid, tannin, and flavonoid compounds. HPLC analysis revealed the presence of catechin as the major compound along with quercetin. Administration of areca nut extract in rats infected with *S. aureus* produced a significant increase in the concentration of WBC but did not affect Hct, Hb, and other cell types. Among the different doses tested, 1000 mg/kg BW was found to be most effective in cellular immunity models. No harmful effects on the liver and kidney functions were observed.

**Conclusion:**

The antioxidant activity of areca nut might be attributed to the presence of catechin and quercetin. Administration of areca nut extract increased the number of WBCs and improved the activity and capacity of macrophages significantly in rats infected with *S. aureus*.

## Introduction

The incidence of infectious disease is increasing, especially in developing countries, thus highlighting the importance of awareness on improving the human body’s immune system mechanism. The act of controlling the immune system is known as immunomodulation. Modulation or changes in immune system refers to the induction, expression, amplification, or inhibition of various phases or parts of the immune response system [1]. Nowadays, the importance of maintaining good function and balance of the immune system has gained increased interest from the community compared to conventional or modern treatments [2]. This is due to the fact that a healthy lifestyle maintained by consuming fresh, nutritious, and natural food has more advantages for the human body compared to the adverse effects of synthetic drugs consumed when immune system suppression occurs. Nevertheless, some diseases may still occur and interrupt the immune system due to imbalance within the body or from the environment, including autoimmune diseases, cancer, bacterial and viral infections, and allergies [3].

Maintenance of oral health and hygiene is one of the fundamental care principles in medically- and immunologically-compromised patients [4]. Oral cavity infection could act as a point of entry for pathogens from which they can spread throughout the body. This is especially true in immunocompromised patients, including patients with malignancy, those with metabolic and immune system impairment, corticosteroid users, or patients undergoing immunosuppressive treatment [4]. The majority of people living in developing countries still use herbal medicines to improve their health. Apart from the lower cost, another factor that draws these patients toward herbal medicines is their belief that the use of synthetic drugs has a risk that may have a detrimental effect on their health [5]. Some community groups in developing countries have the opinion that the use of natural herbal medicine that functions as an immunomodulator is safer than the use of a synthetic drug. To date, no immunomodulatory compound has been developed or synthesized from easily available natural herbs, especially immunomodulatory compounds that can increase the body’s immunity. This presents a vast opportunity for further studies.

Immunomodulatory drugs act by modifying the immune system’s response through increasing (immunostimulatory) or decreasing (immunosuppressive) the production of serum antibodies and immune cells, such as white blood cells (WBCs), macrophages, neutrophils, natural killer cells, and cytotoxic lymphocyte T cells [6]. Immunomodulatory drugs have been developed to selectively inhibit or enhance the intensity, amount, and activity of immune cells [7]. Many studies have demonstrated the immunomodulatory activities of extracts made from plants originating in many parts of the world, such as in India, Taiwan, and China [8-12]. Areca nut is the fruit of one of the palm plants found in various regions of Indonesia, especially in Sumatra, Kalimantan, and Sulawesi [13]. At present, its use as an immunomodulatory drug has not yet been explored.

The aim of the present study was to explore the phytochemical composition and the immunomodulatory potential of areca nut in experimental animals.

## Materials and Methods

### Ethical approval

This study was carried out according to the guidelines for animal experimentation and approved by the Institutional Ethical Committee of Veterinary Medical Teaching Hospital, Bogor Agricultural University (092/KEH/SKE/VIII/2018).

### Sample preparation

Areca nuts were obtained from the *Pinang* plant in Aceh Besar, Indonesia, Botanical Division of Biological Research Center LIPI Cibinong, complete with its roots, stems, leaves, flowers, and seeds in 2018.

### Extraction

The sample used was 5 kg of areca nut (gross weight). Ripe areca nuts were selected from the sample, cleansed from dirt using running water, and dried. The nuts were then shelled and dried in open air and sunlight. Further drying was done using an oven set at a temperature of 50°C. Dried *Simplicia* (unprocessed natural ingredient) was crushed into a fine powder using a blender and then strained with a 20-mesh sieve. The maceration process was conducted by mixing areca nut powder with 96% ethanol diluent. About 4 kg of *Simplicia* was soaked with 96% ethanol in a tightly closed container and stored for 7 days without sunlight, stirring occasionally. Three days later, the extract was strained and dried. Subsequently, 96% ethanol was added to the dried extract and the mixture stirred. The container with the extract was placed in a cool and sunlight-free location for another week. The resulting sediment was then separated from ethanol solution using a rotary evaporator maintained at 30-40°C and then re-concentrated using a water bath until a solid dry powder extract was obtained.

### Preliminary phytochemical screening

The ethanol extract of areca nut was screened for the presence of phytochemical compounds using standard detection methods.

### Alkaloids

Approximately 20 mL of the extract was added to 10 mL of 10% hydrochloric acid (HCl) and ammonia until it reached pH value of 8-9. The mixture was heated for 20 min and cooled, followed by the addition of 5 mL 2% HCl. The aqueous extract was then used to perform the following tests.

#### Mayer’s test

To the filtrate in the test tube I, 1 mL of Mayer’s reagent was added dropwise. The formation of white- or crème-colored precipitate indicated the presence of alkaloids.

#### Dragendorff’s test

To the filtrate in test tube II, 1 mL of Dragendorff’s reagent was added dropwise. The formation of a reddish-brown or orange precipitate indicated the presence of alkaloids.

### Tannins

The ethanol extract of areca nut (0.5-1 mL) was added to 1-2 mL Fe(Cl)_3_ 3%. The formation of blackish-blue precipitate indicated gallate tannin, while a blackish-green precipitate indicated the presence of catechol tannin. In case both the precipitates were observed, separation using 3% formaldehyde: hydrochloric acid (2:1) and heated at 90°C. A red-colored deposit indicated the presence of catechol tannin. A drop of Fe(Cl)_3_ was added to the deposit along with natrium acetate. A color change of the deposit to dark blue indicated the presence of gallate tannin.

### Flavonoids

A 5 mL ethanol extract of areca nut was evaporated until a residue was obtained. Approximately 1-2 mL of methanol was then added to this residue and the mixture heated at 50°C. This was followed by the addition of magnesium and 4-5 mL concentrated HCl. The formation of a red color precipitate indicated the presence of flavonoids.

### Analysis of phenolic compounds using high-performance liquid chromatography (HPLC)

#### Separation and purification of catechins

HPLC mobile phases

The ethanol extract of areca nut was separated using Agilent 1100 HPLC system with Phenomenex Luna 5 µm HPLC 250×4.6 mm column (Torrance, CA) according to the modified method [14]. The reverse-phase separation was then conducted at 30°C using a Waters C18 column (3.9×150 nm) (Waters, USA). The mobile phase consisted of (A) 9% acetonitrile:2% acetic acid with 20 µg/ml ethylenediaminetetraacetic acid (EDTA) and (B) 80% acetonitrile:2% acetic acid with 20 µg/mL EDTAThe gradient was programmed at 100% mobile phase A (4.9.1) for 10 min, followed by a linear gradient for 15 min at 68% mobile phase A, and 32% mobile phase B (4.9.2) for 10 min. Solution C contained 85% phosphoric acid. The flow rate of the mobile phase was 1.0 mL. The binary gradient conditions were: 100% mobile phase A for 10 min, then over 15 min a linear gradient to 68% mobile phase A, and 32% mobile phase B for 10 min. Before the next injection, the device was reset to 100% mobile phase A and allowed to equilibrate for 10 min. The temperature of the column was 35°C ± 0.5°C. The ultraviolet (UV) detector was programmed at 278 nm.

### Preparation of standard solutions

The stock solutions of catechin were prepared by dissolving standards (4 mg/mL) into methanol. Less concentrated solutions were prepared, as needed by dilution with methanol.

### Preparation of samples

About 50 mg of the areca nut extract was transferred into a 100 mL volumetric flask containing 75 mL of methanol, sonicated for 10 minutes, diluted to 100 mL with methanol, and filtered through 0.45 μm membrane filter.

### HPLC analysis

Once the flow rate of the mobile phase and temperature was stable, the column with a blank gradient run was injected with 10 µL each of the standard solutions A, B, and C, followed by an equal volume of diluted areca nut extract. The injection of the mixed standard solutions was repeated at regular intervals. Data collection was done for all peaks in the mixed standards and test extract solution. Data acquisition and processing were performed using a Lab Solution chromatography manager. A 200 µL of the sample was injected into the HPLC. All samples were analyzed in triplicate.

### Separation and purification of quercetin

The mobile phase consisted of 25% acetonitrile in 0.025 M KH_2_PO_4_ (pH 2.4) with a flow rate of 0.9 mL/min (eluent). Detector output was sampled using a Nelson (PE Nelson, Cupertino, CA) Series 900 interface and Nelson integrator software (Model 2600, rev. 5.0). Quantification was based on peak area determined by Nelson. A Hewlett-Packard (Palo Alto, CA) Model 1040 A photodiode array UV-visible detector was used to record the UV spectra of the quercetin. Spectra were recorded at 220-450 nm, 2-nm steps, and sampling interval 1280 ms.

### Experimental animals

Thirty-five adult male Sprague-Dawley rats weighing 200-250 g each were purchased from the Animal House at Veterinary Medical Teaching Hospital. The animals were housed at 23-25°C in a well-ventilated animal house under 12/12 h light/dark cycle and fed with standard pellet diet and tap water *ad libitum*. Bedding material was removed and replaced with fresh paddy husk as often as necessary to keep the animals clean and dry. The rats were quarantined for 14 days before being entered into the experiment.

### Bacteria

*Staphylococcus aureus* was planted in the media and suspended in a peptone broth solution.

### Determination of the effect of areca nut extract on the hematological and biochemical parameters

The plant extract was suspended in normal saline and administered orally for 7 days. The dose-volume was 10 mL. The rats were divided into four equal groups, each group comprising a minimum of five rats: Group I (control) received normal saline; Group II, areca nut extract 500 mg/body weight; Group III, areca nut extract 1000 mg/body weight; and Group IV, areca nut extract 1500 mg/body weight. Two types of blood samples were taken before and after the extract administration from the retro-orbital venous plexus. The first set of blood samples was collected using anticoagulant EDTA tripotassium for erythrogram hematocrit, hemoglobin concentration, red blood count (RBCs), total leukocytic count, and differential cell count. This test was performed using an automatic cell counter. The second set of blood samples was collected without using anticoagulants for blood biochemistry analysis. Blood samples were stored at −20°C. This analysis included the determination of serum glutamate oxalate transaminase (SGOT), serum glutamate pyruvate transaminase (SGPT), urea, and creatinine. Test kits were supplied by BioMerieux-France. After blood analysis was performed, each rat was injected with one dose of intraperitoneal injection of isolated strain of 2 mL *S. aureus* strain (1×10^6^ CFU/mL), then observed for 1 h. The experimental rats were anesthetized using ketamine (25 mg/kg biweekly [BW]) and xylazine (10 mg/kg BW) injection intramuscular.

### Phagocytosis assay

On the 14^th^ day, each rat was injected intraperitoneally with 0.5 mL of *S. aureus* suspension and left for 1 h. The rats were euthanized with ether followed by stomach dissection. Peritoneal fluid was taken using a micropipette and daubed on an object glass and fixed with methanol for 5 min, stained with Giemsa staining and rinsed. Phagocytic activity was determined based on the number of phagocytic cells that actively carried out the phagocytic process in 100 cells. Phagocytic capacity was determined based on the number of *S. aureus* phagocytosed by 50 active phagocytic cells [15].

### Statistical analysis

Qualitative data analysis for HPLC was determined by comparing the catechin and quercetin compounds in areca nut extract and their standards at the same retention time. Quantitative data were calculated based on the level of the regression equation *Y* = *a*+*bx*. The ratio of the total area of concentration and the total content of the polyphenol compounds were calculated in grams of extract. All data in the immunomodulatory analysis were subjected to statistical analysis including the calculation of the mean and standard error (Mean±SE). Significance between hematological, biochemical, and phagocytosis assay parameters in control and treated groups was evaluated by one-way analysis of variance at level p<0.05 using SPSS statistical software (IBM, USA).

## Results

### Qualitative phytochemical analysis

The phytochemical screening indicated the presence of alkaloids, tannins, and flavonoids. Positive tannin compounds produced black-green filtrate, i.e., catechol-tannin compounds. Flavonoid compounds showed a brownish-red color. Alkaloid compounds showed the presence of white-yellowish deposits with Mayer’s reagent and orange deposits with Dragendorff’s reagent, indicating the formation of alkaloid salts. The precipitate formed due to the presence of alkaloids which are nitrogenous base compounds reacting with HCl to form insoluble salts.

### Analysis of catechin and quercetin using HPLC

**Figure-1:**
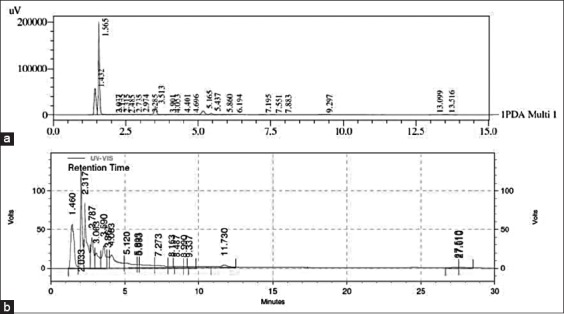
High-performance liquid chromatogram of the areca nut extracts (a) catechin, (b) quercetin.

The crude extract from areca nut was investigated for the presence of catechin and quercetin. In this study, we found that catechin is the major phenolic compound in the areca nut extract (2.79 mg/g). In comparison with catechin, quercetin was found in much smaller quantity in areca nut extract (0.14 mg/g). Figure-1a and b show the HPLC traces of areca nut. The phenolic compounds in the extract were detected by comparing the retention times of the peaks with the standards.

### Analysis of hematology profile

This research is the first study to investigate the ability of areca nut extract to modify the function of the immune cells *in vivo*. The hemogram (Table-1) showed that after 2 weeks post areca nut extract administration and after 1-h post-challenge with *S. aureus*, no significant changes in RBCs count was observed in all groups. On the other hand, the WBC count increased in all three treatment groups (17.49%, 26.99%, and 26.78%, respectively).

**Table-1:** Hematology profile of rats pre- and post-treated with areca nut extract and post-challenge with *Staphylococcus aureus* (Mean±SE).

Parameter	Interval (Days)	Group (mg/kg BW)				Significance

		G1 (Control)	G2 (500)	G3 (1000)	G4 (1500)	
Red blood count cell (×10^6^/mm^3^)	0	7.78±0.22^a^	7.64±0.61^a^	7.52±0.5^a^	7.22±0.41^a^	NS
	14	7.66±0.15^a^	7.64±0.61^a^	7.80±0.27^a^	7.62±0.44^a^	NS
Hematocrit (%)	0	42.94±1.07^a^	42.18±2.33^a^	43.46±2.29^a^	41.14±1.13^a^	NS
	14	42.54±1.17^a^	43.04±1.90^a^	44.40±0.93^a^	42.00±1.38^a^	NS
Hemoglobin (g%)	0	15.48±0.33^a^	14.80±0.82^a^	15.42±0.47^a^	15.06±0.54^a^	NS
	14	15.54±0.79^a^	14.80±0.82^a^	15.28±0.28^a^	14.78±0.58^a^	NS
White blood count cell (×10^3^/mm^3^)	0	9.98±0.70^a^	10.48±1.78^a^	9.86±1.47^a^	10.76±2.35^a^	NS
	14	9.26±1.37^a^	10.88±1.83^a,b^	11.76±0.63^b^	11.74±0.59	*
Lymphocyte (%)	0	68.38±6.63^a^	71.00±7.98^a^	70.68±5.99^a^	67.02±2.86^a^	NS
	14	65.98±4.12^a^	68.20±2.35^a^	71.18±5.81^a^	72.16±3.59^a^	NS
Monocyte (%)	0	9.94±1.59^a^	8.72±4.06^a^	8.14±4.66^a^	9.66±1.36^a^	NS
	14	8.32±0.99^a^	8.72±1.43^a^	8.62±1.59^a^	9.12±1.69^a^	NS
Neutrophil (%)	0	22.42±4.34^a^	20.60±6.65^a^	19.84±1.68^a^	21.64±3.77^a^	NS
	14	24.48±4.63^a^	19.40±5.03^a^	18.62±4.56^a^	17.16±2.80^a^	NS
Basophil (%)	0	0.18±0.13^a^	0.06±0.05^a^	0.08±0.08^a^	0.06±0.05^a^	NS
	14	0.16±0.13^a^	0.06±0.05^a^	0.12±0.04^a^	0.04±0.05^a^	NS
Eosinophil (%)	0	1.08±0.45^a^	0.82±0.46^a^	1.26±0.36^a^	1.02±0.34^a^	NS
	14	1.06±0.46^a^	1.62±1.04^a^	1.46±0.76^a^	1.52±1.13^a^	NS

Means with different superscripts in the same rows are significantly different at p<0.05. NS=Non-significant.

* p<0.05. SE=Standard error, BW=Biweekly

### Analysis of macrophage activity and capacity

**Figure-2:**
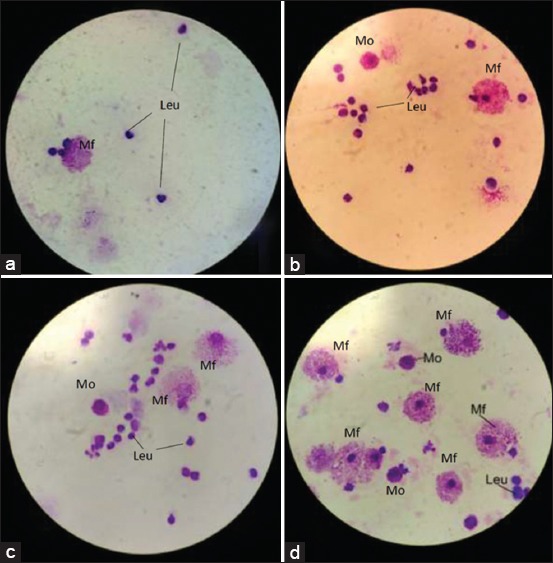
Intraperitoneal fluid in Giemsa staining. (a) The control group showing leukocytes and monocytes, (b) Group II (500 mg/kg biweekly [BW]) showing an increase in the number of leukocytes, and monocytes, (c) Group III (1000 mg/kg BW) showing leukocytes, monocytes, and macrophages, (d) Group IV showing macrophages. Mf=Macrophage phagocyte, Mo=Monocyte, Leu=Leukocyte.

Our study found that areca nut increased the activity and capacity of macrophage. The significant increases were found in all the treatment groups (Table-2). The histopathological findings showed that the number of monocytes, macrophages, and leukocytes increased with an increase in areca nut extract dose (Figure-2).

**Table-2:** Macrophage profile from the intraperitoneal fluid of rats pre- and post-treated with areca nut extract and after 1-h post-challenge with *Staphylococcus aureus* (Mean±SE).

Parameter	Interval (Days)	Group (mg/kg BW)				Significance

		G1 (Control)	G2 (500)	G3 (1000)	G4 (1500)	
Macrophage activity	14	43.86±7.06^c^	71.86±2.61^b^	77.14±5.34^a,b^	80.71±3.35^a,b^	*
Macrophage/100 cells	14	13.71±2.93^b^	16.14±4.41^a,b^	19.43±4.20^a,b^	20.57±4.50^b^	*

Means with different superscripts in the same rows are significantly different at p<0.05. NS=Non-significant.

* p<0.05. SE=Standard error, BW=Biweekly

### Analysis of serum biochemistry

Serum SGPT, SGOT, urea, and creatinine showed a non-significant change in all groups after 1 h of challenge (Table-3). These results showed that areca nut extracts did not cause impaired liver and kidney function after 14 days of treatment and 1-h post-challenge with *S. aureus*.

**Table-3:** Serum biochemistry of rats pre- and post-treated with areca nut extract and post-challenge with *Staphylococcus aureus* (Mean±SD).

Parameter	Interval (Days)	Group (g/kg BW)				Significance

		G1 (Control)	G2 (0.5)	G3 (1)	G4 (1.5)	
SGPT (IU/L)	0	41.10±7.05^a^	41.18±5.69^a^	44.54±7.01^a^	42.12±8.65^a^	NS
	14	47.76±19.11^a^	45.18±12.69^a^	49.72±15.25^a^	49.80±11.68^a^	NS
SGOT (IU/L)	0	121.70±10.10^a^	115.72±11.75^a^	123.98±9.21^a^	131.48±12.64^a^	NS
	14	137.02±17.08^a^	140.92±15.45^a^	142.14±13.89^a^	142.78±13.31^a^	NS
Urea (mg/dL)	0	23.21±3.35^a^	18.78±4.11^a^	22.77±4.66^a^	22.85±1.55^a^	NS
	14	22.55±2.54^a^	18.78±4.11^a^	22.02±3.44^a^	22.91±1.06^a^	NS
Creatinine (mg/dL)	0	0.59±0.07^a^	0.57±0.02^a^	0.61±0.07^a^	0.59±0.03^a^	NS
	14	0.62±0.08^a^	0.57±0.02^a^	0.65±0.05^a^	0.63±0.06^a^	NS

Means with different superscripts in the same rows are significantly different at p<0.05. NS=Non-significant. *p<0.05. BW=Biweekly, SGPT=Serum glutamate pyruvate transaminase, SGOT=Serum glutamate oxalate transaminase

## Discussion

To the best of our knowledge, this was the first study to test the ability of areca nut extract in modulating the immune system in rats. It is a known fact that an increase in age causes a decrease in the capacity of general and specific immune responses, as well as the response toward regulatory signals [16]. Nowadays, immunomodulators from herbal ingredients are widely used to control infectious diseases in addition to antibiotics.

The majority of phytochemical ingredients in areca nut extract are phenolic compounds such as flavonoids, tannins, and alkaloids. We identified the presence of catechin and quercetin through HPLC analysis. Both compounds are known to have very good antioxidant activities; thus, plants containing these components are often used to enhance immunity [17]. Previous studies also have identified several phenolic compounds in areca nut extract, including trimer procyanidin, dimer procyanidin (B1 and B2), catechin, and isorhamnetin 3-O-rutinoside [18]. These compounds have very high antioxidant activities [19,20]. Catechin is proven to have anti-cancer activity in several previous studies [21-24]. Polymerized catechin can suppress the activity of α-toxin produced by *S. aureus* and acts as an effective urease inhibitor in *Staphylococcus saprophyticus* strains [25-27]. This study showed that areca nut also contains quercetin compound, though in lesser quantity. Quercetin is proven to show anti-inflammatory potential in experimental studies in humans and animals [28]. Quercetin could also inhibit cancer cell growth through induction of apoptosis and inhibition of proliferation in gastrointestinal, breast, esophageal, and ovarian cancers [29].

In the present study, areca nut extract was found to increase the WBC count significantly post-challenge with *S. aureus* induction, thus indicating that the extract could stimulate the hemopoietic system. The previous studies showed that areca nut extract could induce calcium signals in three strains of immune cells, namely, lymphocyte B, lymphocyte T, and monocytes [30]. However, areca nut extract could apparently also induce specific immune response toward antigen and could cause *in vivo* inflammatory reaction resulting in immune regulation impairment, leading to areca-related diseases [31]. In our opinion, the study of areca nut as an immunomodulatory drug still causes various effects. It depends on the content of polyphenolic compounds in the areca nut extract which greatly affects the efficacy of the nut.

The result of the activity and capacity of macrophage assay showed an increase in all groups, including the control group. We noted that by increasing the dose of areca nut extract, the activity and the number of macrophages also increased. The areca nut extract probably stimulates the proliferation of macrophages, which in turn leads to the activation of macrophage activity. Further study is needed to find out how the extract leads to an increase in macrophage activity and capacity. However, not only is the effect of the treatment given but also the increase in macrophage activity against *S. aureus* infection might also be caused by the internal factor of the macrophage itself. The previous studies showed that the transmembrane expression of macrophage could suppress the production of nitric oxide and proinflammatory cytokines produced by *S. aureus* in rat’s macrophages [32]. Reports from several studies stated that *S. aureus* could invade and survive in several immune cells such as neutrophils, macrophages, lymphocyte T, fibroblast epithelial cells, endothelial cells, and osteoblasts and this ability is related to bacterial intracellular persistence in certain cells [33-35].

The markers of biochemical examination did not show changes in the liver and kidney in all groups after 2 weeks of treatment and 1 h before and post-challenge with *S. aureus*. This study is in line with the previous studies which revealed that the areca nut consumed by humans does not cause hepatotoxicity in the long term [36,37]. However, another study showed that raw areca nut given for 28 days caused mild hepatotoxicity and nephrotoxicity in mice [38]. Further research is needed to determine the safety limit of areca nut extract for liver and kidney functions.

## Conclusion

Based on the findings from the study, areca nut extract could increase the number of WBCs and improve the activity and capacity of macrophages in rats infected with *S. aureus*. This immunomodulatory ability is probably caused by a phytochemical ingredient in areca nut. Further studies are required to confirm these preliminary findings, to develop an effective immunomodulatory herbal drug with no adverse side effects.

## Authors’ Contributions

AA supervised the present study. LMS designed, coordinated, and performed the experiment. RFH and ZM performed the experiment. LMS analyzed the data and wrote the manuscript. The final manuscript has been read and developed in consultation with all authors.
